# A Novel Prebiotic Blend Product Prevents Irritable Bowel Syndrome in Mice by Improving Gut Microbiota and Modulating Immune Response

**DOI:** 10.3390/nu9121341

**Published:** 2017-12-09

**Authors:** Qian Chen, Yiping Ren, Jihong Lu, Mark Bartlett, Lei Chen, Yan Zhang, Xiaokui Guo, Chang Liu

**Affiliations:** 1 Department of Microbiology and Immunology, Institutes of Medical Science, Shanghai Jiao Tong University School of Medicine, Shanghai 200025, China; xitiruo@sjtu.edu.cn (Q.C.); Lei.Chen@sjtu.edu.cn (L.C.); zhangyan007236@126.com (Y.Z.); xkguo@shsmu.edu.cn (X.G.); 2 Center for Anti-Aging Research, Nu Skin Enterprises, Shanghai 201401, China; yipingren@nuskin.com (Y.R.); jihonglu@nuskin.com (J.L.); 3 Nu Skin Enterprises Anti-Aging Research Center, Provo, UT 84601, USA; mrbartle@nuskin.com

**Keywords:** prebiotics, irritable bowel syndrome, inflammation, visceral hypersensitivity, gut microbiota, prebiotic blend (PB)

## Abstract

Irritable bowel syndrome (IBS) is the most common functional gastrointestinal disorder yet it still lacks effective prevention therapies. The aim of this study is to determine whether a novel prebiotic blend (PB) composed of fructo-oligosaccharide (FOS), galactooligosaccharide (GOS), inulin and anthocyanins could be effective in preventing the development of IBS. We explored the possible mechanisms both in animal and in cells. Post-infectious IBS models in C57BL/6 mice were established and were pretreated with the PB, PB and probiotic strains 8 weeks in advance of infection. Eight weeks after infection, intestinal tissues were collected for assessing histomorphology, visceral sensitivity, barrier function, pro-inflammatory cytokines expression and proteomics analysis. Fecal samples were also collected for microbiota analysis. The pro-inflammatory cytokines expression in Caco-2 cells were evaluated after co-incubation with PB and *Salmonella typhimurium* 14028. The results showed that PB significantly decreased the pro-inflammatory cytokines both in infected Caco-2 cells and PI-IBS models. The loss of body weight, decreased expression of tight junction protein Occludin (OCLN), and changes of the microbiota composition induced by infections could be greatly improved by PB intervention (*p* < 0.05). The proteomics analysis revealed that this function was associated with Peroxisome proliferator-activated receptor (PPAR)γ pathway.

## 1. Introduction

Functional gastrointestinal disorders (FGD) are recognized by physiological abnormalities that contain motility disturbances, increased visceral sensitivity and altered central nervous system function without organic illness. Irritable bowel syndrome (IBS) is the most common type of functional disorder characterized by abdominal pain and discomfort and associated with altered bowel function [1,2]. Based on the symptoms, IBS may be characterized by a predominance of constipation (IBS-C) or diarrhea (IBS-D) or by mixed bowel habits (IBS-M), and unsubtyped IBS (IBS-U) [2,3]. The global prevalence of IBS is 11.2% with the incidence rates at over 20% in England, America, Greece and Pakistan, and rates of 10–15% in China, Canada and Australia [4,5]. The high prevalence together with the reduced quality of life in patients suffering from IBS imposes a significant negative burden on both patients and society. Meta-analyses demonstrated a six-seven-fold increased risk of developing IBS after a gastroenteritis episode, making gastrointestinal infections probably the strongest known risk factor for the development of IBS [6]. Such incidences are defined as post-infectious IBS (PI-IBS). As acute infective gastroenteritis (IGE) affects millions of persons per year, particularly travelers to the developing world, PI-IBS poses a potential large burden of illness [7]. For the past decades, different psychological profiles and illnesses are known as risk factors for persistent post-infectious symptoms: Both depression and anxiety are considered as risk factors [8]. Patients with PI-IBS show some similar symptoms with those of non-infectious origin [9].

IBS is also considered as a “brain-gut disorder” [10]. Abdominal pain, visceral hypersensitivity, altered motility of gut and increased intestinal wall permeability are all involved in the pathophysiology of IBS [11]. Preliminary studies revealed that alterations in immune activation of the mucosa and the dysbiosis of gut microbiota may contribute to the development of IBS. Indeed, the role of gut microbiota was regarded as a key factor influencing the “brain-gut communication”. Gut microbiota has been confirmed to play various roles in maintaining host health such as metabolizing drugs, producing essential vitamins, and preventing the colonization of pathogens [12]. A growing number of research demonstrates that the diversity, stability and metabolic activity of the gut microbiota are altered in most IBS patients compared with healthy individuals [13,14,15]. It is worth mentioning that the disturbed microbiota is one of the most important pathologic factors of PI-IBS. A marked shift in microbiota following acute gastroenteritis might be long lasting responsible for the prolonged symptoms of PI-IBS [16]. 

Due to the heterogeneity of IBS, it is difficult to design meaningful regimens to benefit all patients. Various therapies such as peppermint oil, lubiprostone, linaclotide, antibiotics, diet intervention and soluble fiber are in use [10]. However, all these therapies appear to be symptomatic. Since the importance of gut microbiota in IBS pathogenesis has been identified, the manipulation of the microbiota is emerging as an attractive therapeutic option for this disease [17]. Interventions of probiotics, prebiotics, synbiotics and antibiotics are examples of such strategies used to modulate the gut microbiota in IBS patients [18,19,20,21,22,23,24,25,26,27,28]. Most of these trials were proven to be effective in relieving the symptoms of IBS. The prebiotics, defined as “non-digestible food ingredients that beneficially affect the host by selectively stimulating the growth and/or activity of one or a limited number of bacterial species already resident in the colon, and thus attempt to improve host health” is also known as “functional food”. These prebiotics escape absorption in the small intestine and enter the large intestine where they provide nutrients for beneficial bacteria [29]. This concept definition has been further expanded as “a substrate that is selectively utilized by host microorganisms conferring a health benefit” [30]. Several prebiotics belong to the group of non-digestible carbohydrates: monosaccharides, disaccharides, oligosaccharides, polyols and so on. However, these therapeutic approaches are only used to relieve the symptoms of patients with IBS rather than cure the disease. Few studies exist that examine the effects of prebiotics in modulating gut microbiota for the prevention of the development of IBS. This study constitutes the investigation of a novel prebiotic product named “PB”, composed of fructo-oligosaccharide (FOS), galactooligosaccharide (GOS), inulin and anthocyanins in this context. We designed this study to verify whether PB or the symbiotics containing PB and certain probiotic strains could be effective in preventing IBS. Moreover, we explored the possible mechanism of such treatment in a post-infectious mouse model and in a colonic epithelial cell line. 

## 2. Materials and Methods

### 2.1. Bacteria Cultures

*S. typhimurium* 14028 was grown in fresh Luria Broth media overnight under 37 °C until they reached the late exponential phase. The bacteria culture suspension was diluted, and the optical density was determined. Probiotic strains used in this study include *Lactobacillus acidophilus* NCFM and *Bifidobacterium lactis* HN019 in the form of lyophilized powder (Pharmanex^®^ ProBio, Lot. DP22451, supplied by Center of Anti-aging Research, Nu Skin Enterprises, Shanghai, China). 

### 2.2. Prebiotic-Containing Product

The PB (supplied by Center for Anti-aging Research, Nu Skin Enterprises, Shanghai, China) was composed of GOS plus FOS, inulin and anthocyanins in the form of freeze-dried powder. The powder was suspended in saline before treatment.

### 2.3. Caco-2 Cell Treatment

**Figure 1:**
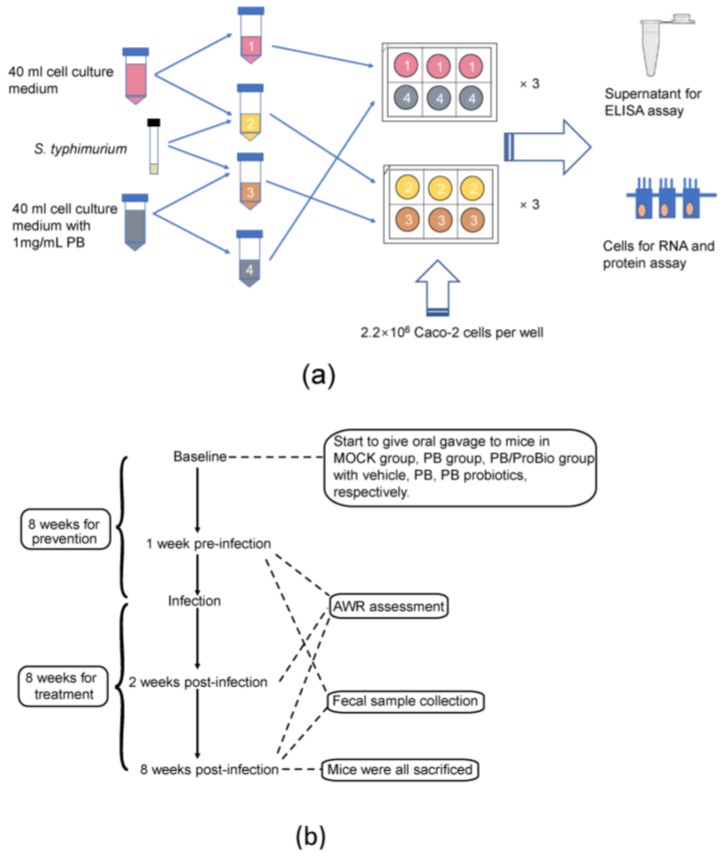
The schematic diagram of (**a**) Caco-2 cells experiment and (**b**) mice model experiment.

Caco-2 cells were grown in 55 cm^2^ petri dishes (CORNING) with DMEM medium (Gibco, Carlsbad, CA, USA) supplemented with 2 mM l-glutamine (Gibco), 0.2 mM 15% fetal bovine serum (Gibco), and 100 Units/mL Streptomycin and Penicillin (Gibco), maintained at 37 °C in a 5% CO_2_ incubator (Thermo Scientific, Waltham, MA, USA). Then the cells were seeded to 6-well plates (COSTAR) in a density of 2.2 × 10^6^ cells per well until reaching 80–100% confluence. Starved Caco-2 cells were incubated for 12 h in the cell culture media without FBS supplementation. *S. typhimurium* was added to cell culture media and inoculated to the Caco-2 cells in 6-well plates at the multiplicity of infection (MOI) 10:1. PB was supplemented to the culture media at a dose of 1 mg/mL. After stimulation times of 0.5 h, 1 h, and 2 h, the supernatants were collected for enzyme-linked immunosorbent assay (ELISA) detections. Total RNA of the cells is extracted. The workflow of Caco-2 cell experiment is shown in Figure 1a; all the experiments were repeated triplicated.

### 2.4. Animal Treatment and Experimental Diets

Four-week-old female specific pathogen-free (SPF) C57BL/6 mice used in this study were obtained from Animal Laboratory Center of Shanghai Jiao Tong University School of Medicine (Shanghai, China) and kept in a laminar flow cabinet in the experimental animal room. The experimental procedure was approved by the Animal Welfare committee of Shanghai Jiao Tong University School of Medicine. Before the trial, 60 mice were randomly divided into 4 experimental groups (15 mice per group in 3 cages), among which 3 groups were given a gavage of saline (MOCK group), PB (1.26 mg/g body weight, PB group), PB and probiotics (1.26 mg/g body weight PB plus 3.0 × 10^7^ CFU/mouse of both *Lactobacillus acidophilus* NCFM and *Bifidobacterium lactis* HN019, PB/ProBio group), respectively. The treatment was conducted each day until they were sacrificed. The last group was defined as control group. This procedure is shown in Figure 1b. All animals were housed in a 12 Light, 12 Dark cycle. Body weights were measured weekly. Stool samples were collected both at the baseline and 8th week post infection. Total DNA was extracted from the stool with QIAamp^®^ Fast DNA Stool Mini Kit (Qiagen, Hilden, Germany). 

### 2.5. Trichinella Spiralis Infection

After 8-week treatment, mice in the first 3 groups were infected with *Trichinella spiralis* larvae. The infective larvae were obtained from the muscle of C57BL/6 mice infected for 30 days in advance. The infected mice were humanely sacrificed, skinned, and the muscles containing encysted larvae were minced and digested in 2.5% pepsin A and 1.5% HCl at 37 °C for 20 h. The isolated infective larvae were washed for several times, filtrated with 70 μm filter, and resuspended in normal saline. Mice in the experimental groups were infected by the oral administration of 350–400 larvae in 0.2 mL of saline, while mice in the control group received the same volume of vehicle.

### 2.6. Abdominal Withdrawal Reflex (AWR) Scores Assessment

Visceral hyperalgesia to colorectal distention (CRD) was assessed at the 8th week post infection (PI) by AWR scores. A catheter (6-Fr, 2 mm external diameter) was inserted rectally into the descending colon of mice. After adapting for 0.5 h, colorectal distention was performed in a stepwise fashion. Each 20-s distention was followed by a 30-s resting period. Each level of distention (0.15, 0.2, 0.3, and 0.35 mL) was repeated three times and the score is mean value. The balloon was deflated and withdrawn after assessing AWR. The AWR score was assigned as follows: 0, no behavioral response to colorectal distention; 1, brief head movement followed by immobility; 2, contraction of abdominal muscles; 3, lifting of abdomen; 4, body arching and lifting of pelvic structures [31,32].

### 2.7. Sample Collection and Processing

All mice from each group were humanely sacrificed (ether anesthesia and cervical dislocation) after 8 weeks’ infection. Intestinal samples taken from the ileum (30 cm distal to the pylorus) and colon (distal to the caecum) were flushed with normal saline to remove gut contents. A 1 cm long sample from each intestinal tissue was fixed overnight in 4% paraformaldehyde and embedded in paraffin for histological analysis. Two pieces of 1.5 cm long samples of the intestine tissue was immediately preserved in liquid nitrogen and stored in cryogenic refrigerator for subsequent RNA extraction and protein assay, respectively. 

### 2.8. Histological Analysis

Ileum tissues fixed in paraformaldehyde were embedded in paraffin and cut into 5-μm-thick sections. To deparaffinize, the sections were immersed in xylene at 56 °C twice for 20 min, and hydrated with ethanol (twice with 100%, once with 95%, and once with 75% ethanol) for 5 min. The tissues were processed routinely for hematoxylin and eosin (H&E) histology. 

### 2.9. Immunohistochemistry (IHC) Detection

Antibodies against Occludin (OCLN) (1:1000 dilution; Abcam, Cambridge, UK) were used for IHC. Dry tissue sections of 6 μm thickness at 60 °C for 20 min. Slides underwent dewaxing and hydration with sequential dimethylbenzene washes of 20 min, 100% ethanol washes of 10 min, then sequential ethanol washes of 5 min, which started with 95% ethanol, followed by 80% and finishing with 75% ethanol. Wash slides with Phosphate buffered saline (PBS) for twice. The antigen was retrieved by citric acid buffer water bath heating at 95 °C for 20 min, and then restored at room temperature. Block endogenous peroxidase by incubating 20 min in 3% H_2_O_2_ and wash slides with PBS. Block non-specific binding sites with 5% BSA for 20 min. The sections were probed with rabbit monoclonal antibodies against occludin (1:1000 dilution; Abcam) at 4 °C overnight. After washing the slides with PBS, the tissues were probed with biotinylated secondary antibody for 20 min, and reveal the resulting peroxidase activity by incubating the slides with diaminobenzidine (DAB) for 7 min. Counterstain for 1 min with haematoxylin. Dehydrate slides with sequential ethanol washes of 5 min, each starting with 75%, followed by 80%, 95% and 100% ethanol wash, finishing with a dimethylbenzene washes. 

### 2.10. Quantitative Real-Time Polymerase Chain Reaction (qRT-PCR) Assay

Total RNA of Caco-2 cells, colon tissues from each mouse were extracted using Trizol solution. cDNA were synthesized with RevertAid First Strand cDNA Sythesis Kit (Thermo Scientific). The transcript levels of IL-1β (interleukin-1β), IL-6, IL-10, IL-17, TNF-α (tumor necrosis factor-α), IFN-γ (interferon-γ) were determined by real-time quantitative PCR reaction with 7500 Fast Real-Time PCR System (ABI, Waltham, MA, USA), GAPDH (glyceraldehyde-3-phosphate dehydrogenase) was set up as internal reference. Each qPCR reaction was performed in duplicate, and the expression level was determined as the mean value. Primer sequences were listed in Table 1.

### 2.11. Western Blot Analysis

RIPA (radio-immunoprecipitation assay) lysis and extraction buffer (Beyotime, Shanghai, China), 0.1~1 mM PMSF (Beyotime) were used for the protein extraction from intestinal tissues and the cells lysis. The protein concentration was determined with Pierce™ BCA Protein Assay Kit (Thermo Scientific). Proteins were separated by 10% sodium dodecyl sulfate polyacrylamide gel electrophoresis (SDS-PAGE), and then transferred to a nitrocellulose blotting membrane (GE Healthcare, Chicago, IL, USA) which have been blocked by 5% skim milk. The membrane was incubated with the primary antibodies of β-actin and the target protein at 4 °C overnight. Then the secondary antibodies were added and incubated at room temperature for 1 h. After exposure with SuperSignal West Pico Chemiluminescent Substrate (Thermo Scientific), protein bands were shown and mugged by LAS-4000 MINI.

### 2.12. Fecal Microbiota Analysis

The total DNA was extracted from fecal samples. The 16S rDNA high-throughput sequencing was performed by Realbio Genomics Institute (Shanghai, China) using the Illumina HiSeq PE250. Variable regions V3–V4 on 16S rDNA genes of bacteria were amplified with forward primer F341 5′-ACTCCTACGGGRSGCAGCAG-3′ and reverse primer R806 5′-GGACTACVVGGGTATCTAATC-3′. The raw paired end reads were assembled by pandaseq with overlap nucleotides. Then, the reads were quality-filtered. The raw data were then subjected to a quality control procedure using UPARSE. The qualified reads were clustered to generate operational taxonomic units (OTUs) at the 97% similarity level using Usearch. Principal components analysis (PCA), heatmap analysis, Bray-Curtis similarity cluster, and species abundance analysis were performed using R.

### 2.13. Proteomics Analysis

Preparations of 100 μg protein from colon were made in triplicate for each group. For each sample, proteins were precipitated with ice-cold acetone, and then were re-dissolved in 100 μL TEAB (tetraethyl-ammonium bromide). Then proteins were tryptic digested with 1:50 sequence-grade modified trypsin (Promega, Madison, WI, USA), and the resultant peptide mixture was labeled with iTRAQ (isobaric tags for relative and absolute quantitation). The peptide mixture was separated at high pH, and twelve fractions were collected and each fraction dried in a vacuum concentrator for the next step. Then nano-HPLC (high-performance liquid chromatography)-MS (mass spectrum)/MS analysis was conducted at low pH. Scaffold (version Scaffold_4.7.5, Proteome Software Inc., Portland, OR, USA) was used to validate MS/MS based peptide and protein identifications. Scaffold Q+ (version Scaffold_4.7.5, Proteome Software Inc.) was used to quantitate peptide and protein identifications.

### 2.14. Statistical Analysis

The significance of the differences in AWR scores between the 3 PI-IBS groups and the health control group was initially determined by regular two-way ANOVA and Tukey test. The significance of the differences in cytokine amounts between experimental groups was initially determined by one-way ANOVA. Multiple comparisons were performed using the Scheffe’s post hoc test. The significance analysis of bacteria genera among the 4 groups was detected by Mann-Whitney test with Benjamini and Hochberg multiple testing adjustment, and Bonferroni correction. Results were considered statistically significant at * *p* < 0.05, ** *p* < 0.01, *** *p* < 0.001, **** *p* < 0.0001.

## 3. Results

### 3.1. Pro-Inflammatory Cytokine Profiles in Caco-2 Cells and Animal Models

**Figure 2:**
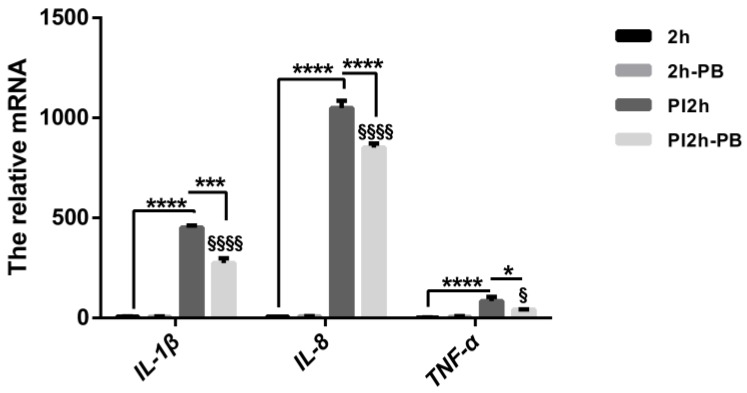
mRNA expression of pro-inflammatory cytokine profiles in Caco-2 cells after relative treatment for 2 h. All data are presented as mean value ± SEM. * *p* < 0.05, *** *p* < 0.001, **** *p* < 0.0001. § Different from 2h-PB, § *p* < 0.05, §§§§ *p* < 0.0001.

**Figure 3:**
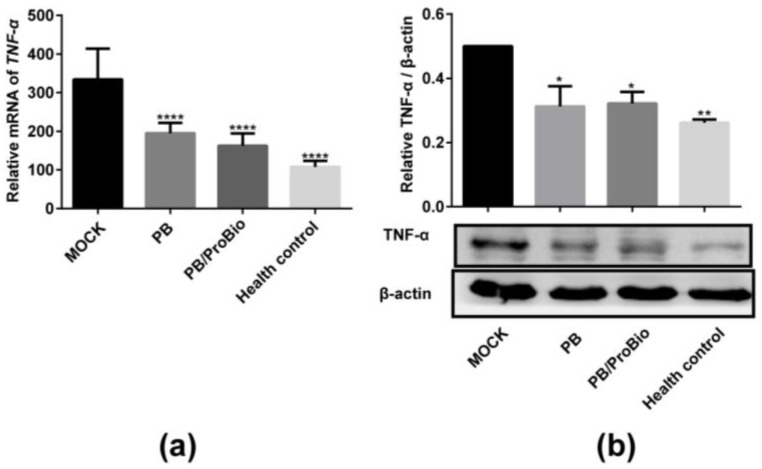
The effect of prebiotic blend (PB) and probiotics on modulating *Tnf-a* expression in colon tissues. The relative mRNA of each group was shown by mean value ± SEM, *n* = 8 for each group. (**a**) The relative mRNA of *Tnf-α* in the 4 groups. **** *p* < 0.0001. (**b**) Upper panel: Relative intensity of TNF-α protein expression calculated as the ratio of TNF-α vs. β-actin. * *p* < 0.05, ** *p* < 0.01. Lower panel: Western blot analysis of TNF-α in MOCK, PB, PB/ProBio, and Health control groups.

The mRNA levels of cytokines in Caco-2 cells measured by qRT-PCR revealed a dramatic increase of *IL-1β*, *IL-8* and *TNF-α* after infection with *S. typhimurium* for 2 h. These cytokine levels were shown to decline significantly in the Caco-2 cells incubated with PB. Likewise, mRNA expression of these pro-inflammatory cytokines did not show significant activation after PB treatment of the Caco-2 cells (Figure 2). In colon tissues, the mRNA level of *TNF-α* was markedly increased in the *Trichinella spiralis* infected groups, but were decreased significantly in both the PB and the PB/ProBio groups. Protein level detection by western blot showed the same tendency (Figure 3). But the expression level of *IL-10* almost unchanged among the 4 groups.

### 3.2. Symptoms in PI-IBS Model

**Figure 4:**
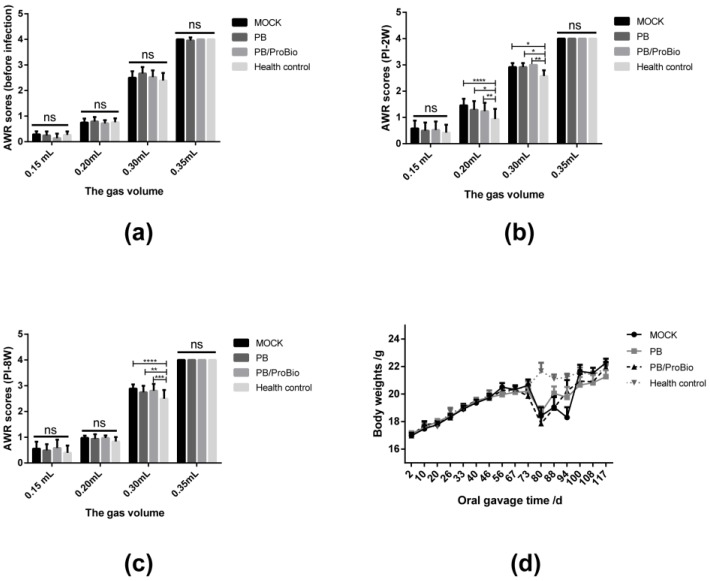
The change of body weights and Abdominal Withdrawal Reflex (AWR) scores in the 4 groups. AWR scores at different levels of distension in (**a**) one-week pre-infection, (**b**) 2 weeks post-infection, and (**c**) 8 weeks post-infection. *n* = 15 in each group. (**d**) The change of body weights. * *p* < 0.05, ** *p* < 0.01, *** *p* < 0.001, **** *p* < 0.0001.

On the 2nd week post infection, the body weights of mice decreased sharply in infected groups, and recovered slowly in the next week (Figure 4d). Furthermore, the intestinal tissues were damaged severely on the 1st week post infection and restored after the acute infection period (Figure S1). No significant differences in AWR scores were shown among the 4 groups before infection. These scores were significantly higher in the 3 infected groups at distention volumes of 0.20 and/or 0.30 mL. On the 8th week post-infection, the AWR score could be reduced by PB or PB/ProBio treatment which indicates that the visceral hypersensitivity is lower in these two groups, but not recovered completely like the normal state (Figure 4a–c).

### 3.3. Tight Junction Protein in PI-IBS Mice

**Figure 5:**
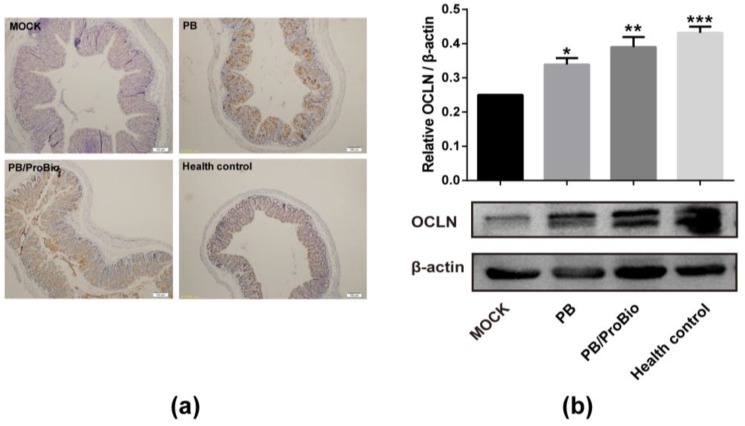
Protein expression of Occludin (OCLN) in different groups. (**a**) Immunohistochemistry analysis of the OCLN in colon. (**b**) Upper panel: Relative intensity of OCLN protein expression calculated as the ratio of OCLN vs. β-actin. Lower panel: Western blot analysis of OCLN in MOCK, PB, PB/ProBio, and Health control groups. * *p* < 0.05, ** *p* < 0.01, *** *p* < 0.001.

The expression of tight junction protein OCLN was examined by western blot analysis. The result showed that the OCLN expression in the MOCK group was weakened compared with that of the health control group. Elevated expression of OCLN were observed both in PB and PB/ProBio groups (Figure 5a). IHC analysis of OCLN revealed a similar tendency (Figure 5b). 

### 3.4. PPARγ Expression

**Figure 6:**
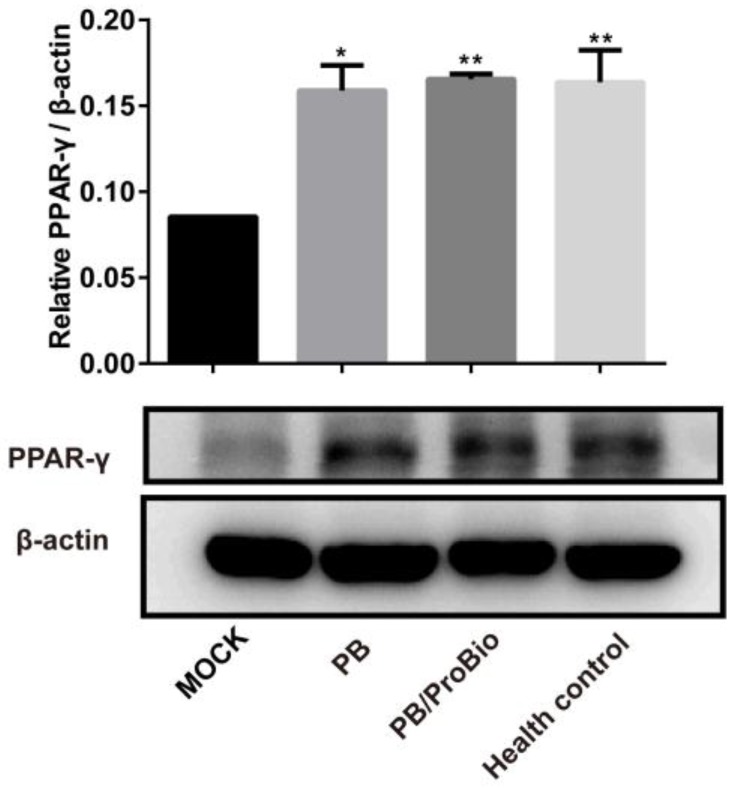
The expression level of PPARγ in the 4 groups. All data were presented as mean ± SEM. (*n* = 3). Upper panel: Relative intensity of PPARγ protein expression calculated as the ratio of PPARγ vs. β-actin. Lower panel: Western blot analysis of PPARγ in MOCK, PB, PB/ProBio, and Health control groups. * *p* < 0.05, ** *p* < 0.01.

The proteomics analysis detected 5403 proteins in each group. When comparing these proteins between the MOCK group and the other 3 groups, the majority of these proteins were different. A small portion of these different proteins that increased or decreased more than 1.2 folds are exhibited in the heatmap (Figure S2). It was discovered that PPARγ signaling was changed significantly (*p* < 0.05) when compared MOCK group with PB group using KEGG pathway analysis. The expression of PPARγ in colon tissues were confirmed using western blot analysis, which elucidated the expression of PPARγ decreased in the MOCK group compared with the health control, but increased in the PB group and the PB/ProBio group significantly in protein levels (Figure 6).

### 3.5. The Alteration of Microbiota

**Figure 7:**
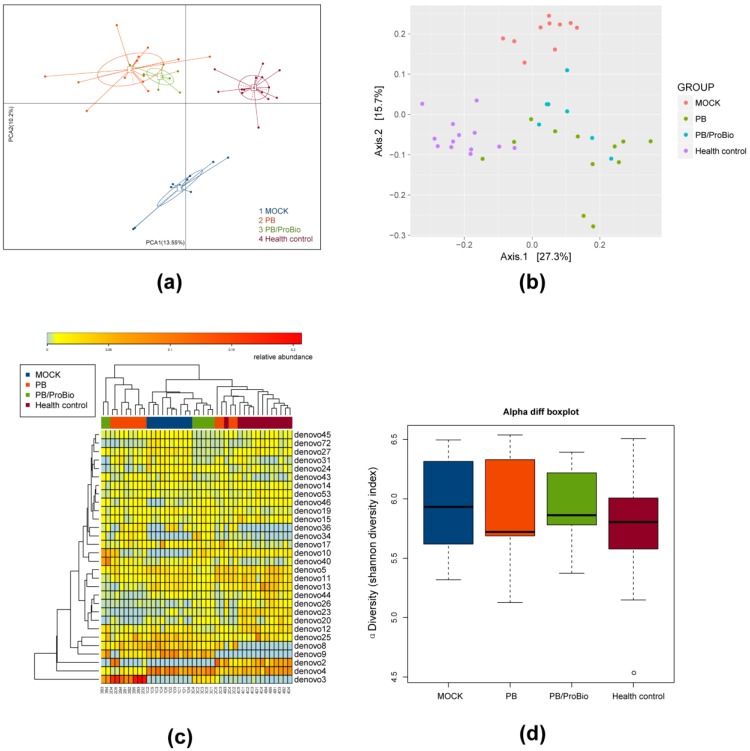
Gut microbiota changes and α-diversity in different groups. (**a**) Principal components analysis (PCA) plot showing microbiota communities cluster with different treatment. (**b**) Non-Metric Multidimensional Scaling (NMDS) plot based on Bray-Curtis distances of the fecal microbiota. Each data point represents a fecal sample from these groups, with the corresponding labels indicated on the right. (**c**) Heatmaps and clustering of individual gut microbiota samples for taxonomic composition. (**d**) Shannon diversity.

**Figure 8:**
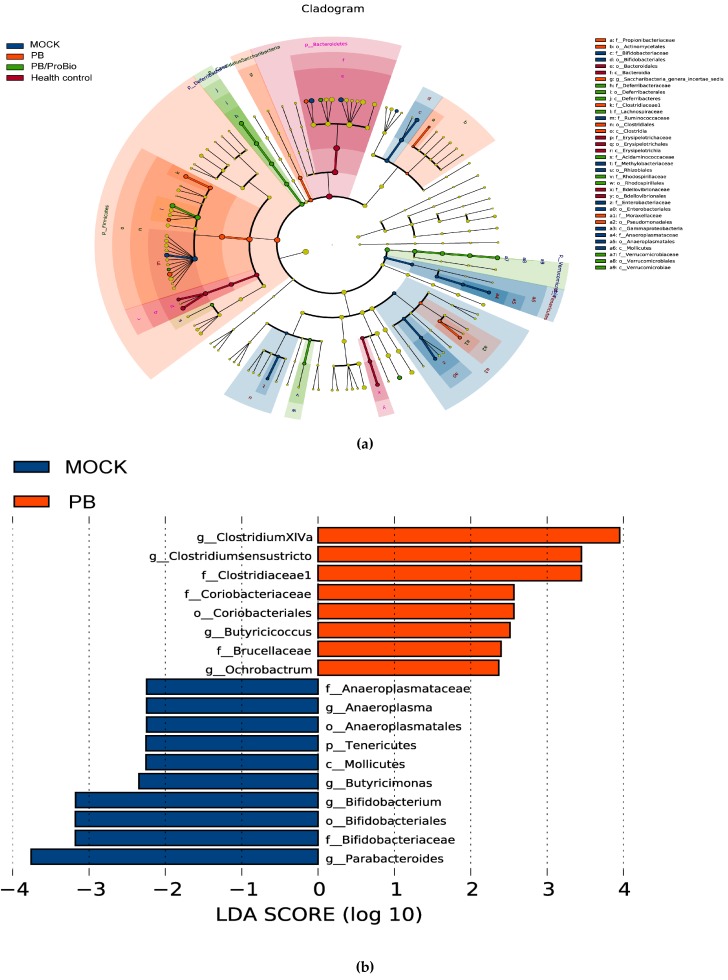

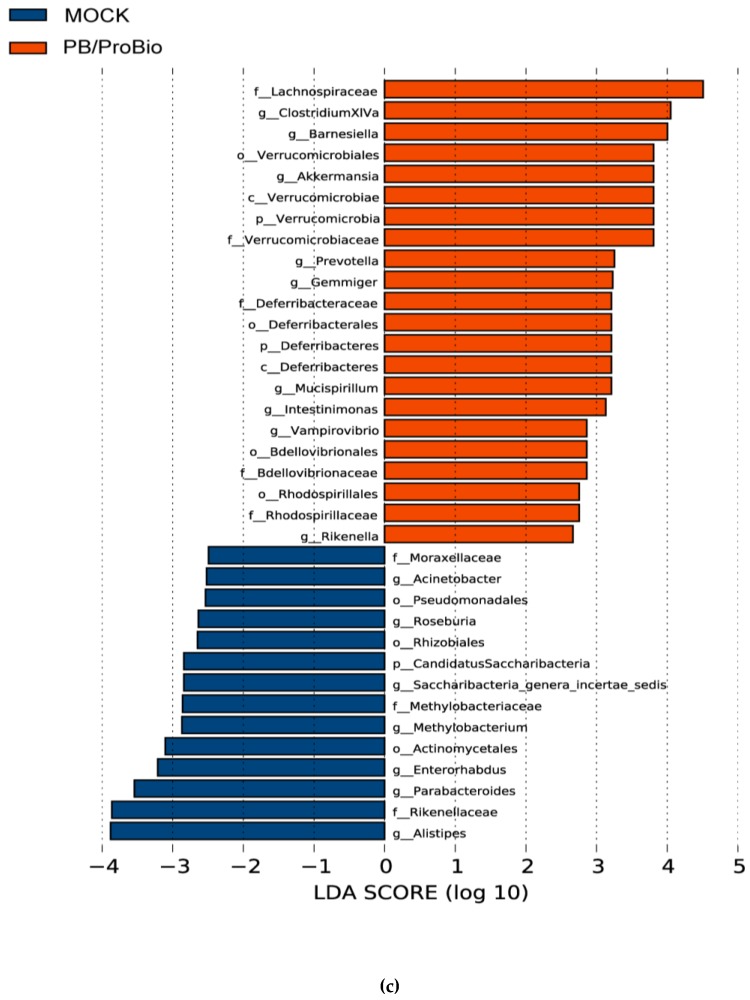

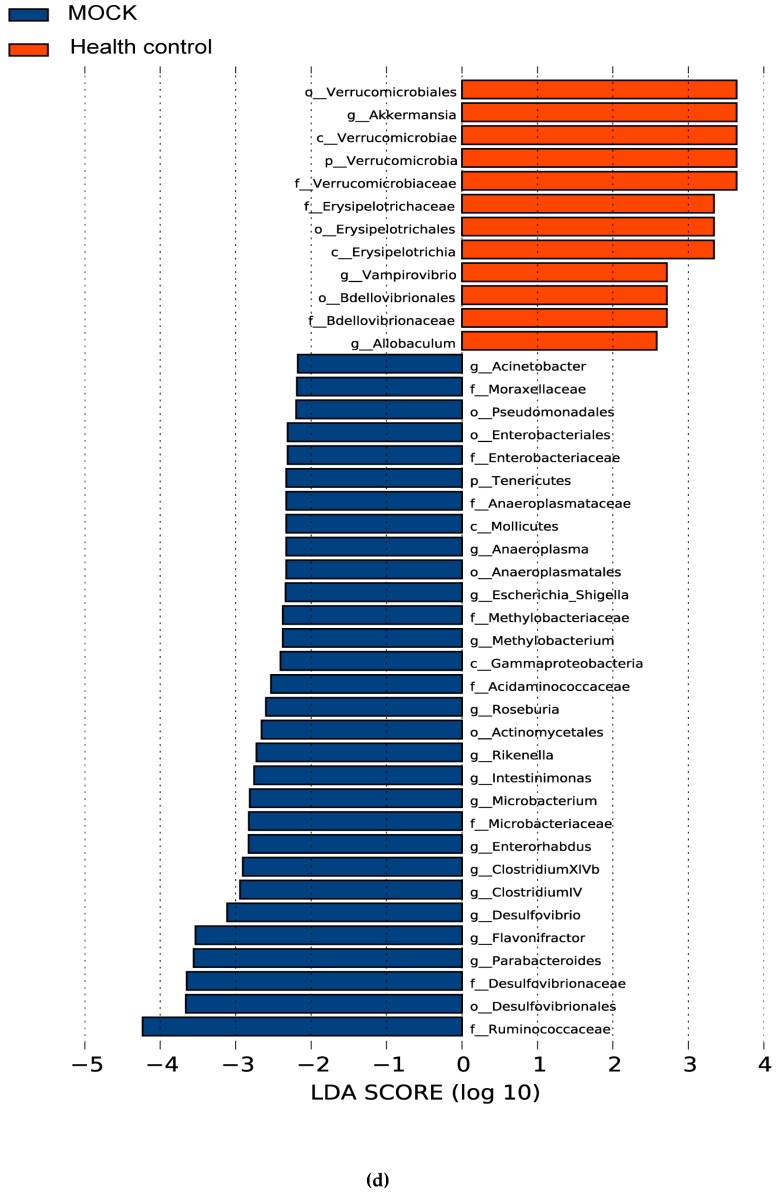
The comparison of the fecal microbiome project of MOCK group to other groups. All data of the taxonomic information in four groups were analyzed with Metaphlan2 program, the cladogram (**a**) was derived from LEfSe analysis (http://huttenhower.org/galaxy/). Linear discriminant analysis (LDA) of the fecal microbiota in MOCK group and PB group (**b**), MOCK group and PB/ProBio group (**c**), MOCK group and Health control (**d**). A *p*-value of <0.05 and a score ≥2.0 were considered significant in Kruskal–Wallis and pairwise Wilcoxon tests, respectively. The horizontal straight line in the panel indicates the groups means.

PCA and Bray-Curtis similarity cluster analysis together with a heatmap cluster based on OTU abundance were performed to provide an overview of the gut microbiota composition of the three PI-IBS groups and health control group (Figure 7a–c). No significant differences in α-diversity of microbiota were shown among all groups (Figure 7d). The overall microbiota composition feature of the MOCK group was significantly different from that of the heath control. Meanwhile, the presence of PB or PB/ProBio improved this alteration. After Bonferroni correction in the microbiota analysis, only a few genera tend to have significant differences among different groups. Linear discriminant analysis (LDA) showed that the abundance of the bacteria genera of *Vampirovibrio* and *Akkermansia* decreased in MOCK group, while increasing in the PB/ProBio groups. The relative abundance of the genera *Clostridium*XIVa, *Clostridium_sensu_stricto*, *Prevotella*, *Butyricicoccus*, *Ochrobactrum*, *Barnesiella*, *Gemmiger*, *Mucispirillum*, *Intestinimonas*, and *Mucispitillum* increased in PB group or PB/ProBio group compared with the MOCK group. The abundance of the genera *Acinetobacter*, *Roseburia*, *methylobacterium*, *Parabacteroides*, *Rikenella*, *Intestinimonas*, *Microbacterium*, *Enterorhabdus*, *Anaeroplasma*, *Roseburia*, *Clostridium* IV, *Clostridium* XIVb, *Desulfovibrio*, and *Escherichia/Shigella* increased significantly in the MOCK group in contrast with health control. Except for the former four bacterial genera, the amount of the other bacteria genera decreased relatively in the MOCK group compared with PB and PB/ProBio groups (Figure 8a–d). 

## 4. Discussion

**Figure 9:**
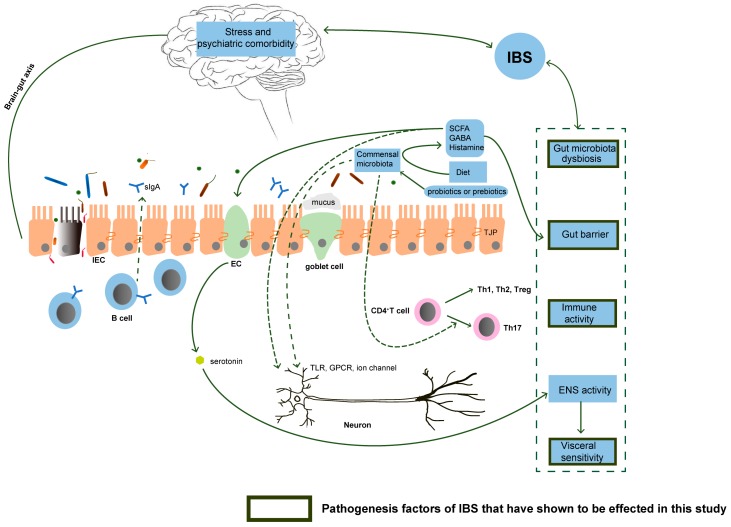
The pathogenesis factors related to irritable bowel syndrome (IBS) that can be affected by PB and PB/ProBio. The gut microbiota dysbiosis was improved by PB and PB/ProBio dramatically. Probiotics have been shown in many experimental models to stimulate regulatory T cells. A variety of prebiotics and probiotics are known to decrease immune-mediated activation in IBS [70,81]. PB and PB/ProBio exerted anti-inflammatory effect both in vitro and in vivo. Many available probiotics can reinforce the intestinal barrier function via enhance the expression of tight junction proteins, promote mucus secretion and host cell antimicrobial peptides, and directly release antimicrobial factors [82]. PB and PB/ProBio are shown to increase tight junction protein expression in this study. Enteric neurones express Toll-like receptors displaying sensitivity to microbial metabolites, and thus the enteric nervous system (ENS) has the capacity to respond to microbes [83]. SCFAs, the metabolites of normal microbiota, are able to stimulate sympathetic nervous system and mucosal serotonin release. Microbiota can influence ENS activity by producing molecules that can act as local neurotransmitters, such as GABA and histamine and by generating a biologically active form of catecholamines in the lumen of the gut [84]. The activation of ENS is responsible for visceral hypersensitivity, which can be suppressed by PB and PB/ProBio. Abbreviations: SCFA, short chain fatty acid; EC, enterochromaffin cells; IEC, intestinal epithelial cells; TJP, tight junction protein; GABA, gamma amino butyric acid; TLR, toll like receptor; GPCR, G protein couple receptor; ENS, enteric nervous system.

Irritable bowel syndrome is one of the most prevalent lower gastrointestinal tract disorders in which the etiological factors are very complicated (Figure 9). IBS symptoms can be triggered by acute or chronic enteric infections and can persist for weeks, months, even years [33,34]. PI-IBS accounts for 4~39% of all IBS cases [35,36]. Therefore, a post-infectious model is an applicable model for IBS studies. We established a successful PI-IBS mouse model in this study. Eight weeks after *Trichinella spiralis* infection in mice, the damaged intestinal epithelium layer and lamina propria had already recovered (Figure S1). Nevertheless, visceral hypersensitivity and low grade inflammation still existed, combined with a decreased level of expression of intestinal tight junction protein. These phenomena are consistent with former studies on IBS models [31]. 

Treatment of IBS with currently available medications usually is targeted to relieve the individual symptoms, such as constipation, diarrhea, and abdominal pain [10]. Specific probiotic strains and prebiotics known as fructo-oligosaccharides, disaccharides, monosaccharides and polyols have been proven as effective therapies for IBS treatment by different mechanisms (Figure 9) [37,38,39,40,41]. Nevertheless, the preventive effect of prebiotics or probiotics for IBS is little known. Several studies have reported a role of anthocyanins in the modulation of inflammation and oxidative stress and were shown to help to reduce the risk of chronic diseases [42,43]. Compared with live probiotic bacteria, the application of our prebiotic product is safer [44]. The aim of present research is to investigate the preventive function of this novel supplementary compound in IBS. Our results showed that the clinical manifestation of the PI-IBS model could be significantly improved by PB pre-intervention. In this study, mice lost body weight after oral gavage of *Trichinella spiralis* larvae compared with the health controls. However, the body weight of mice treated with PB and probiotics recovered faster. 

Visceral hypersensitivity is an important pathological mechanism and physiological marker in IBS patients. This visceral hypersensitivity may account for the symptoms of urgency, bloating, and abdominal pain experienced by patients with IBS [45]. In our study, AWR scores were increased significantly in the PI-IBS groups in response to medium pressures on the colorectum, but did not differ from healthy mice at low or high pressure. When the extension volume was 0.15 mL, the pressure on the colorectum was too low to cause different visceral sensations in all the mice. When 0.35 mL gas was injected into the colorectum, AWR scores of almost all the mice were at level 4 mL, an indication that the gas volume had reached the threshold of visceral pain. Mice in the first 3 PI-IBS groups showed significantly higher AWR scores, which indicated an increase of visceral sensitivity in mice after infection. However, after pre-treatment with PB and PB/ProBio, mice did not show marked differences with the MOCK group, while treatment with PB notably reduced the significance of the difference compared to the MOCK vs. health control after both two and eight weeks’ post-infection. This result demonstrated that the successfully established PI-IBS model can be improved with PB intervention. 

In a clinical study containing 111 patients with IBS and 162 health controls, the combination of a “high producer” TNF genotype and a “low producer” IL-10 genotype was more prevalent in patients with IBS [46]. Moreover, divergent results for cytokine levels have been reported. In some studies, the levels of TNF and IL-6 secreted by PBMCs (peripheral blood mononuclear cells) from IBS patients, while in the normal range, were higher than levels secreted by stimulated PBMCs from health controls in other studies [47,48,49]. In order to determine whether PB ameliorated inflammatory status in PI-IBS mice and Salmonella stimulated Caco-2 cells, we detected the expression of pro-inflammatory mediators both in colon tissues and Caco-2 cells. In Caco-2 cells, the activation of *TNF-A*, *IL-1B* and *IL-8* induced by *S. typhimurium* could be inhibited by PB. As far as we know, prebiotics serve as “colonic food” for colonic bacteria [30]. But they can suppress inflammation in a microbiota-independent way, in which these components can stimulate NF-κB (nuclear factor kappa B) pathway and directly regulate host kinome [50,51]. In the animal model, we found that the mRNA and protein levels of TNF-α in the MOCK group increased significantly with an almost invariant level of IL-10 (not shown), while declining in the PB or PB/ProBio prevention groups. Both the in vitro and in vivo results suggested PB can attenuate inflammation both in the Caco-2 cells and the IBS mice model. 

The most obvious feature of the intestinal barrier consists of a single layer of mucosal epithelial cells that are interconnected by tight junctions that allow the passage of small molecules [11]. Twelve to fifty percent of IBS patients have been reported to have decreased intestinal permeability [52]. An ever increasing number of studies support the concept that the increased intestinal permeability is relevant to enhanced activity of the immune system and food allergy [53]. Fermentable fiber has been shown to reduce the recovery time and improve tight junctions in the gut of piglets with bacterial infection induced enteritis [54]. Our molecular analysis of the tight junction related proteins demonstrated a decrease of the OCLN expression in PI-IBS mice. Eight-week supplementation of PB dramatically reversed this change which showed increased intestinal permeability, indicating the role of PB in protecting the integrity of the intestinal epithelium layer.

Dysbiosis of gut microbiota is considered to play a critical role in the development of IBS. Emerging data suggest that the diversity, stability and metabolic activities of the fecal microbiota are altered in most patients with IBS compared with health controls [13,14,15]. According to previous studies, the cluster of fecal microbiota in a PI-IBS group is clearly distinct from that of the health control. From a therapeutic perspective, certain probiotic strains, prebiotic products, even antibiotics such as rifaximin which may contribute to modulate microbiota were applied in IBS treatment. In PB, the main ingredients are FOS, GOS, inulin and anthocyanins which were all reported separately to modulate intestinal microbiota. Inulins have been reported to stimulate growth or activity pf beneficial bacteria and suppress the level of harmful bacteria selectively [55,56]. Recent studies also showed that anthocyanins from blackberries could alter the composition and diversity of microbiota [57]. In the PB pretreatment group, the composition of the microbiota was more similar to health controls which suggest that the PB could help maintain the homeostasis of gut flora. At the genus level, we detected a high proportion (>5%) of *Barnesiella* in all groups. The level of the genus *Barnesiella* significantly decreased in the PI-IBS group while this change was greatly reversed in PB pre-treatment group. *Barnesiella*, a genus of the family of *Porphyromonadaceae*, order *Bacteroidales*, was one of the most abundant genera detected in the mouse intestine. In recent studies, many new sequences have been isolated from human and animal intestine that can be identified as the genus *Barnesiella*. Nevertheless, it still remains unclear how the abundance of *Barnesiella* is linked to inflammatory diseases of the gastrointestinal tract [57]. Some reports shed light on the function of Barnesiella, reporting that Barnesiella bacteria contribute to the elimination and protection against vancomycin-resistant Enterococcus faecium colonization [58]. The abundance of *Barnesiella* is indicated to correlate with the level of several immunoregulatory cells. The higher the levels of *Barnesiella* in the colon, the more marginal zone B cells and invariant natural killer T cells enumerated in the spleen and liver [58,59]. The direct association between a change in Barnesiella and the resistance to arthritis was demonstrated in mouse models [60]. In the development of colitis in IL-10^−/−^ mice, higher levels of a *Barnesiella* phylotype correlated with lower activity levels of the disease [61]. Such observations indicate that the genus *Barnesiella* is beneficial to human health by protecting the intestinal tract from pathogen infections and have a role in immunomodulation. Significantly higher levels of *Barnesiella* were observed after consumption of PB/ProBio and indicated anti-inflammatory effects in IBS prevention. Many reports have shown that the relative abundance of Coliform bacteria, *Veillonella*, *prevotella*, *Lactobacillus*, *Parasporobacterium*, *Firmictes*, mainly *Clostridium* cluster XIVa and *Ruminococcaceae* increased significantly in patients with IBS, together with a reduction in the relative abundance of Bacteroidetes [11]. In the human, commensal Clostridia start to colonize the intestine of breastfed infants during the first month of life and populate a specific region in the intestinal mucosa in close relationship with intestinal cells. Clostridia are thus strongly involved in the maintenance of overall gut function [62]. Commensal Clostridia play an important role in the metabolic welfare of colonocytes by releasing butyrate as an end-product of fermentation which exert multiple beneficial effects on regulating mammalian metabolism [63,64,65,66]. Several reports have revealed that Clostridia appeared to be involved in the promotion of immunological development in the colon [64]. Notably, in experimental mice models, it was indicated that elevated levels of *Clostridium* clusters XIVa in mice can lead to resistance to allergy and intestinal inflammation [67]. In this study, the increase of *Clostridium* XIVa and *Clostridium sensu stricto* in the PB/ProBio group or PB group might be associated with protective roles in IBS. In previous studies, members of the genus *Akkermansia* have been suggested as biomarkers for a healthy intestine [68,69,70]. In the present study, the abundance of *Akkermansia* in the PI-IBS model significantly decreased. Nevertheless, addition of probiotic bacteria instead of PB itself can greatly upregulate the level of *Akkermansia*. This result might suggest that live bacteria could stimulate the abundance of *Akkermansia* other than prebiotics. Bifidobacterium, shown to have health promoting properties, have been shown to be decreased in most cases of IBS [12,71,72,73]. One study found that inulin-type fructans and GOS promote the growth of lactobacilli or bifidobacteria [73]. However, in our study, there is no evidence to prove that PB or PB/ProBio can increase the amount of Bifidobacterium. 

PPARs contain a three-member sub family including PPARα, PPARβ/δ, and PPARγ. The PPARγ is a nuclear lipid-activatable receptor controlling the expression of genes involved in lipid metabolism and adipocyte differentiation [74]. It is expressed not only in intestinal epithelial cells (IECs) but also in intestinal macrophages and T cells [75]. PPARγ was reported to inhibit the production of inflammatory cytokines in different cell types by interfering with TLR4-dependent signaling pathway [76]. A clinical study comparing a healthy control group with Crohn’s disease patients found that the expression of PPARγ in the intestinal tissue of patients with ulcerative colitis (UC) was significantly decreased [77]. Moreover, it has been shown that prebiotics such as oligosaccharides can reduce proinflammatory cytokines in intestinal Caco-2 cells, and that this reduction is dependent upon the activation of PPARγ [78]. Using a proteomic analysis, compared with PI-IBS model, PPARγ would be predicted to be activated in the PB pretreatment group in the colon. Thus, we further verified by western blot analysis. This result confirmed the previous research indicating that the PPARγ plays a crucial role in regulating inflammatory conditions and in inhibiting inflammation in intestinal tract, which may prevent the development of PI-IBS.

The PB product is a mixture of naturally sourced prebiotics and anthocyanins. Compared with probiotic bacteria, the consumption of prebiotics is safer to human health, though they might not be completely safe, which we will discuss later. The key safety aspects for use of probiotic strains include systemic infections, deleterious metabolic activities, excessive immune stimulation in susceptible individuals and gene transfer [79]. In our study, the prebiotic product PB itself has the same ability to prevent IBS in the mouse model as the supplementation of probiotic bacteria. On the other hand, the prebiotics are believed to be more stable to store and transport. 

Although in this study, the prebiotics blend can improve the symptoms of IBS efficiently, and many studies have shown that prebiotics can help with the resistance to gastrointestinal infections [80], there are several researches about how certain prebiotics increase the potential infection possibility to certain pathogens. I M J Bovee-Oudenhoven et al. have shown that rapid fermentable inulin and FOS decreased resistance of rats towards salmonella as early as 2004 [81]. Moreover, it was revealed that mice fed a high fiber diet exhibited a 10- to 100-fold increase in colonization of Shiga toxin-producing *Escherichia coli* O157:H7 and had 25% greater mortality relative to the low fiber diet-fed group [82]. However, in our study, the PB product could inhibit the salmonella induced inflammation in vitro and *Trichinella spiralis* induced inflammation in vivo. Further researches might need to be conducted to prove whether the PB product will increase the risk of infection to other pathogens. 

Taken together, the results of this study demonstrated that a novel prebiotic product PB could attenuate the inflammatory response both in Caco-2 cells and in a PI-IBS mouse model. Pre-treatment with PB in mice significantly decreased the severity of IBS symptoms which provides biological plausibility for the usefulness of this prebiotic product. Importantly, we have shown that the infection-induced gut bacterial shifts could be reversed by the PB product compared with the negative control. We also found that PPARγ signaling has a direct connection with the anti-inflammatory effects of PB, but the specific signaling pathway cannot be determined from this study. Prebiotics are inexpensive, non-invasive and safe to use. Therefore, our results provide a foundation for the application of this PB to prevent IBS in human populations. Further research is needed to confirm preventive efficacy in IBS patients. Well-designed studies are required to understand the exact mechanism of this function. Collectively, as a novel prebiotic product, daily dietary interventions of PB might be a candidate for the prevention of IBS. On the flip side, some prebiotics take a role as a potential threat for certain gastrointestinal infections. Before the clinical use of this product, sufficient randomized controlled trials (RCTs) and necessary safety assessments are supposed to be conducted. 

## 5. Conclusions

The results of this study demonstrated that a novel prebiotic-containing product PB was able to attenuate the inflammatory response both in Caco-2 cells and the PI-IBS model. Pre-treatment of PB in mice significantly decreased the severity of the IBS symptoms and modulated the gut microbiota in the PI-IBS model. This provides biological plausibility to the usefulness of this prebiotic product as a preventive measure for gastrointestinal dysbiosis and dysfunction. 

## Figures and Tables

**Table 1:** Quantitative Real-Time Polymerase Chain Reaction (qRT-PCR) primers sequences used in this paper.

Genes		Sequences (5′-3′)	NCBI Gene ID
*β-actin* (mouse)	Forward	AGTGTGACGTTGACATCCGT	11461
	Reverse	TGCTAGGAGCCAGAGCAGTA	
*TNF-α* (mouse)	Forward	GAGGCCAAGCCCTGGTATG	21926
	Reverse	CGGGCCGATTGATCTCAGC	
*PPAR-γ* (mouse)	Forward	GGAAGACCACTCGCATTCCTT	19016
	Reverse	GTAATCAGCAACCATTGGGTCA	
*GAPDH* (human)	Forward	ATGGGGAAGGTGAAGGTCG	2597
	Reverse	GGGTCATTGATGGCAACAATATC	
*IL-1B* (human)	Forward	GAAATGCCACCTTTTGACAGTG	3553
	Reverse	TGGATGCTCATCAGGACAT	
*IL-8* (human)	Forward	GACCACACTGCGCCAACAC	3576
	Reverse	CTTCTCCACAACCCTCTGCAC	
*TNF-α* (human)	Forward	GAGGCCAAGCCCTGGTATG	7124
	Reverse	CGGGCCGATTGATCTCAGC	

## Supplementary Materials

The following are available online at www.mdpi.com/2072-6643/9/12/1341/s1. Figure S1: H&E staining of ileum tissues after infection for 1 week, 2 weeks, 4 weeks, 6 weeks. Figure S2: Hierarchical cluster analysis (HCA) to find discrete groups between PB group, PB/ProBio group, and health control vs. with varying degrees of (dis)similarity in a data set represented by a (dis)similarity matrix. The pie chart described the amount of proteins determined by proteomics analysis.
