# Supplementary figures and images for: A Novel Prebiotic Blend Product Prevents Irritable Bowel Syndrome in Mice by Improving Gut Microbiota and Modulating Immune Response
Source: Nutrients. 2017 Dec 9;9(12):1341. doi: 10.3390/nu9121341 (PMC5748791; doi:10.3390/nu9121341)

Figure S1

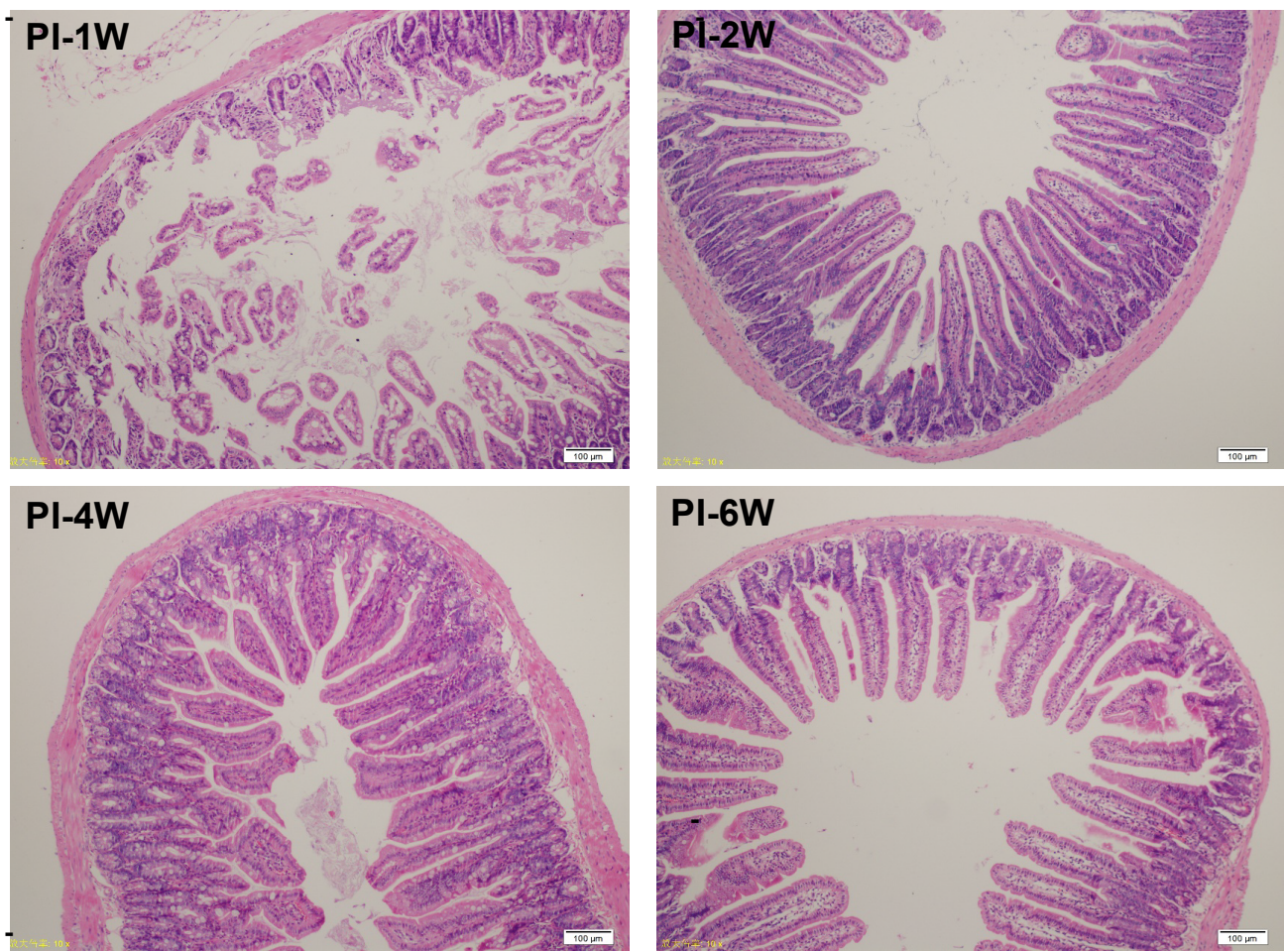

Figure S2

A

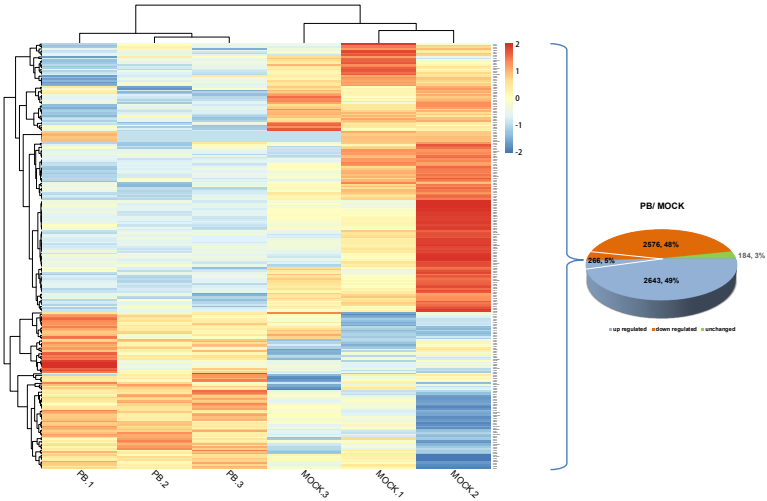

B

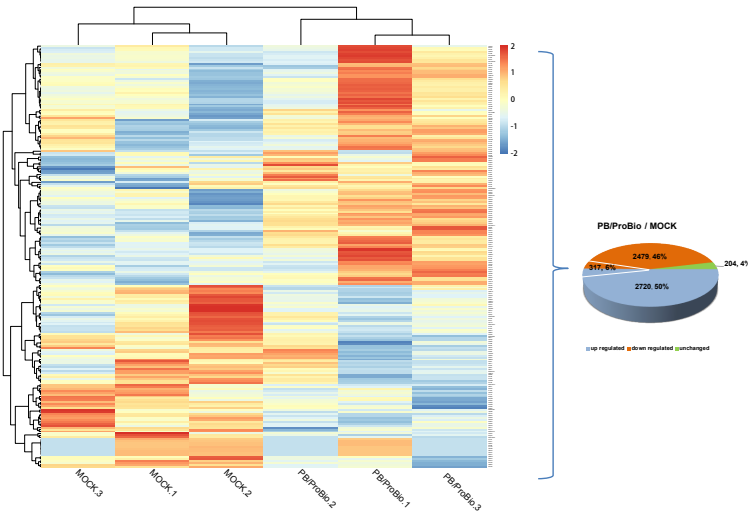

C

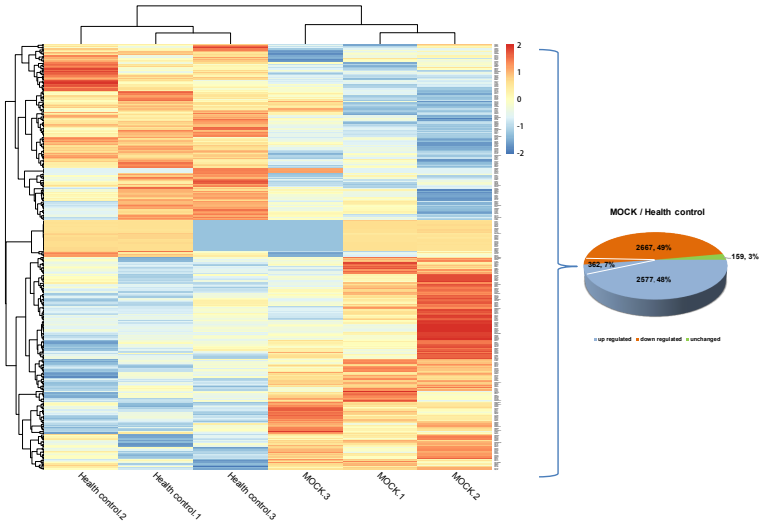

Supplement: Supplementary file 1 [file nutrients-09-01341-s001.pdf]
